# Biases in Perceiving Positive Versus Negative Emotions: The Influence of Social Anxiety and State Affect

**DOI:** 10.3390/vision9040092

**Published:** 2025-11-01

**Authors:** Vivian M. Ciaramitaro, Erinda Morina, Jenny L. Wu, Daniel A. Harris, Sarah A. Hayes-Skelton

**Affiliations:** 1 Department of Psychology, University of Massachusetts Boston, Boston, MA 02125, USAharrisda@udel.edu (D.A.H.); sarah.hayes@umb.edu (S.A.H.-S.); 2 Department of Epidemiology, University of Delaware, Newark, DE 19716, USA

**Keywords:** faces, emotion, perceptual bias, state affect, social anxiety

## Abstract

Models suggest social anxiety is characterized by negative processing biases. Negative biases also arise from negative mood, i.e., state affect. We examined how social anxiety influences emotional processing and whether state affect, or mood, modified the relationship between social anxiety and perceptual bias. We quantified bias by determining the point of subjective equality, PSE, the face judged equally often as happy and as angry. We found perceptual bias depended on social anxiety and state affect. PSE was greater in individuals high (mean PSE: 8.69) versus low (mean PSE: 3.04) in social anxiety. The higher PSE indicated a stronger negative bias in high social anxiety. State affect modified this relationship, with high social anxiety associated with stronger negative biases, but only for individuals with greater negative affect. State affect and trait anxiety interacted such that social anxiety status alone was insufficient to fully characterize perceptual biases. This raises several issues such as the need to consider what constitutes an appropriate control group and the need to consider state affect in social anxiety. Importantly, our results suggest compensatory effects may counteract the influences of negative mood in individuals low in social anxiety.

## 1. Introduction

Faces are one of the most important sources of visual stimuli for informing and mediating social engagement. A face conveys information along multiple dimensions, allowing us to glean basic information about a person, such as perceived age, gender, race, relative attractiveness, and identity, e.g., [1,2]. Furthermore, faces express emotions that provide rich and dynamic insight into another person’s mental state. As a social species, our ability to read, interpret, and act, based on the emotional expressions of others, is a critical skill, e.g., [3]. Conversely, a breakdown in the ability to accurately interpret and respond to the emotional status of others can impair social relationships and therefore place individuals at higher risk of social isolation.

One such example, of the breakdown in the ability to interpret emotions, is the effect of social anxiety disorder, a significant mental health condition affecting approximately 12% of the U.S. population [4]. Social anxiety disorder is characterized by persistent fear and anxiety in social or performance situations [5]. Some suggest that social anxiety is an evolutionarily adaptive mechanism that evolved as our social systems came to rely on skills in decoding social signals as threatening or non-threatening in order to facilitate group living [6,7]. Prominent models of social anxiety suggest that this disorder is characterized, in part, by perceptual and information processing biases; see [8,9]. According to these models, when an individual with social anxiety enters or anticipates a social situation, they form a negative mental representation of how the audience perceives them. At the same time, the individual may selectively attend to information that confirms this negative perception of the self and of the audience’s perception of them. This negative bias, coupled with post-event rumination, can lead to an overestimation of the likelihood of negative consequences, thereby leading to avoidance and a continuation of this anxious cycle.

One of the central features, common to these models of social anxiety, is a negative bias towards the perceived audience or social stimuli in general. Individuals with social anxiety tend to interpret neutral social stimuli as more negative compared to individuals without significant social anxiety see [10] for a review. Theory suggests this initial negative bias of social stimuli exacerbates the negative evaluation of one’s own performance, which then increases the likelihood of rumination and avoidance and, subsequently, increases an individual’s anxiety. Given that this initial negative bias likely maintains social anxiety [11], it is important to understand how negative biases operate and manifest so that future work can help reduce or counter such patterns. This is especially important given that not all studies find evidence for an enhanced negative bias in individuals high in social anxiety, as discussed in detail below.

### 1.1. Biases in Social Anxiety

When one considers the underlying mechanisms that may drive enhanced fear and negative evaluation in social anxiety, the literature has focused on biases in memory, attention, and perception of emotional cues. Some studies further suggest a combination of such mechanisms. For example, an initial attention bias toward threat information [10,12,13,14] and an avoidance of positive cues [15] may later manifest as avoidance of threatening features such as the eyes [16], thus supporting an early vigilance for detection and later avoidance hypothesis [17]. Additionally, evidence suggests that social anxiety mediates the influence of emotional cues on attention, altering the processing of low-level visual stimuli [18]. Emotional cues of fearful vs. neutral faces were found to be more effective at directing exogenous attention in individuals high in social anxiety, benefitting performance on orientation discrimination, as well as enhancing perceived contrast, with a reported PSE difference between cued and uncued locations ranging from 0.1 to 0.2 (log units) [18].

**In terms of perceptual biases, social anxiety has been characterized by a tendency to perceive emotional expressions as more negative.** Socially anxious individuals also have elevated sensitivity to negative expressions of emotion, demonstrating lower thresholds for identifying and detecting faces conveying anger and disgust, e.g., [19,20]. Individuals high in social anxiety show more frequent detection of angry facial expressions [21], enhanced recognition of negative facial expressions relative to positive expressions [22], and tend to rate negative facial expressions more negatively compared to individuals low in social anxiety [23,24].

A multitude of studies have demonstrated a bias for perceiving neutral or ambiguous emotions as more negative; for a review, see [25]. In particular, individuals high in social anxiety are more likely to judge neutral or ambiguous expressions as threatening, perceiving anger or disgust, unlike non-socially anxious controls, who judge ambiguous expressions as non-threatening, perceiving sadness [19,26]. This bias in emotional processing contributes to the cycle of social anxiety, as this ambiguity allows for open interpretations, and socially anxious individuals, already prone to fear negative evaluations, would be biased to perceive social cues as signs of disapproval [8]. In an emotion interpretation task, individuals high in social anxiety are more likely to interpret neutral faces as threatening, whereas individuals low in social anxiety interpret the same emotionally ambiguous faces as neutral [27]. Similarly, in an emotion card-sorting task, individuals high in social anxiety show greater accuracy sorting angry facial expressions and are more likely to interpret neutral faces as angry, further demonstrating a bias to perceive ambiguous emotions as more negative [28]. This bias in perception can also be understood as a reduced bias to perceive ambiguous emotional information as non-threatening. Individuals high in social anxiety not only interpret neutral information as more threatening but are also less likely to interpret neutral information as benign [29,30]. In sum, several underlying mechanisms may enhance negative biases when individuals high in social anxiety view the emotional information conveyed by a face.

It is worth noting, however, that a number of studies have shown inconclusive results regarding threat-related interpretation bias for emotional faces in individuals high in social anxiety. For example, some studies have used morphed emotional faces, manipulating affective intensity along a continuum, and found only partial evidence of a negative bias for detecting negative emotions at the lower emotional intensities in face morphs [31,32]. Other studies investigating the perception of ambiguous facial stimuli failed to find any difference in perception as a function of social anxiety [33,34]. Some of these underlying discrepancies between studies may arise due to differences in the populations studied in terms of other factors such as cognitive empathy or the presence of other comorbid disorders.

One important factor which may explain discrepancies across studies in finding perceptual biases for ambiguous faces is state affect, a short-term and transient emotional state of arousal [35]. According to Mathews and Mackintosh [36], increases in fear or state affect can decrease an individual’s threshold in judging stimuli as more threatening, and this phenomenon might be pronounced and more frequent in individuals with higher levels of trait anxiety. Indeed, several studies have examined participants with differing levels of trait anxiety, for example, high and low social anxiety, in induced state anxiety situations to investigate the interaction between state and trait anxiety [29,37,38].

### 1.2. Influence of Trait vs. State Affect

Many experimental paradigms only provide “snapshots” (i.e., when measurements are taken) of dynamic cognitive processes [39], making it important to also consider state, or mood, ratings that correspond to those moments in time. Furthermore, state and trait ratings have been shown to have differential stability over time and convey unique information [40], as well as activating distinct neural circuits, e.g., [41]. The literature on affect variability highlights the importance of considering state-level variations in within-person affect even when the focus is on a certain trait-level characteristic [42].

In terms of negative biases, past work shows that greater state anxiety is associated with quicker reaction times to respond to threat-related words [43]. In addition, placing individuals high in social anxiety in a stressful situation decreases their accuracy in interpreting facial expressions [44] and can lead to faster reaction times in avoiding angry faces [45]. Interestingly, inducing state anxiety has also been associated with increased attentional bias to threat regardless of trait anxiety severity [38]. Thus, a growing body of work speaks to the interrelations of state and trait affect. Specifically, state anxiety can alter the effects of trait anxiety on cognitive processing [46,47]. Highlighting the importance of considering both trait and state affect, past work shows that individuals high in trait social anxiety are slower to identify low-intensity angry faces under conditions with no stress; however, under stress (i.e., state stress), they respond more quickly [48]. Thus, it is hypothesized that increases in negative state affect may enhance ratings of threat (i.e., perception of threat), whereas trait anxiety may modulate attention in response to the threat [49].

The interaction of state and trait affect has also been shown to influence the accuracy of interpreting emotional stimuli. Individuals high in social anxiety are better at interpreting low-intensity angry faces when sorting emotional faces following a threat manipulation [28]. They also show a higher rate of misclassifying neutral faces as angry. This is in contrast to non-socially anxious individuals who have a higher rate of misclassifying angry faces as neutral [28]. Of note, the response patterns in these examples were observed when state affect was experimentally induced. Yet, current state affect, even when not induced, has also been shown to influence perceived emotion in non-clinical cohorts [50,51] and to moderate attentional bias patterns in clinical cohorts [52].

In line with this perspective, more recent systematic reviews highlight the use of real-time measurements of individuals’ behaviors and psychological states, including negative and positive affect, using techniques such as ambulatory assessments to emphasize the importance of studying social anxiety in a more ecological way [53]. Specifically, Fernández-Álvarez and colleagues’ [53] review of 70 studies showed that increases in negative affect consistently co-occur with heightened social anxiety symptoms; however, negative affect alone is not sufficient to distinguish normative from clinical social anxiety. Thus, it is crucial to test how naturally occurring, momentary negative affect at the time of testing interacts with trait social anxiety to bias perception. Such insight may explain when certain symptoms of social anxiety become maladaptive, manifesting as perceptual biases that reinforce negative interpretations of social cues.

Notably, not all influences of state affect are negative. Ecological momentary assessment shows that individuals with increased social anxiety, although experiencing higher baseline distress, can benefit from positive daily events. In fact, experiencing intense positive experiences are associated with increased happiness, reductions in avoidance of social interactions, and a greater sense of belonging in these individuals [54].

Taken together, a growing body of publications in the literature suggests that trait anxiety does not operate in isolation and highlights the importance of considering the moderating effect of contextual influences and state affect, both current and induced, in studies of perceptual bias in social anxiety. More specifically, relatively few studies have directly examined how non-induced state affect moderates cognitive biases in social anxiety. Addressing this gap is critical to resolving the inconsistencies in the literature and refining theoretical models and practical implications of social anxiety.

### 1.3. The Current Study

Given the key importance of negative bias in social anxiety and the important contribution state affect may have on perceptual bias, the current study extends previous work by (1) using a parametric approach to quantify the magnitude of perceptual bias for individuals high and low in social anxiety and (2) examining the contributions of current, non-induced, state affect in modifying the relationship between social anxiety and perceptual bias. In our task, participants rated male and female faces varying in the extent of angry, happy, and neutral expressiveness. To quantify perceptual biases, we measured each participant’s point of subjective equality (PSE). The PSE is the point along a continuous stimulus range of emotion (in our case, angry to happy) where an observer cannot discriminate between emotional categories—judging a face equally often as happy or angry. This measure is advantageous, since there are individual differences in which faces are considered ambiguous, even in non-clinical cohorts. For example, Webster and colleagues [55] reported large individual differences in category boundaries such that sex boundaries along a male–female continuum of faces are shifted towards the participant’s own sex, with similar biases for race. Given the large variation in what each individual perceives as a neutral face, it is unclear what face to present as an emotionless, ambiguous, standard.

We hypothesized that (1) there would be a stronger negative perceptual bias to perceive faces as angry in individuals high compared to low in social anxiety, and (2) this negative bias would be stronger in individuals displaying greater negative state affect before the experiment began, especially in individuals high in social anxiety. Of note, given that depression is often comorbid with social anxiety [56], and that depression can contribute to negative mood [57], our study design limited the influence of depression by only including participants scoring in the lower range on a depression scale.

#### Transparency and Openness

Data and analysis code are available on Open Science Framework via this link: https://osf.io/bjypt/. The study design and analysis were not preregistered. Here, we include baseline data collected as part of a bigger study on adaptation to emotional faces. Data from a subset of these adaptation studies have been reported in related work [58]. Sample size was determined for these adaptation studies from which this baseline data was collected.

## 2. Materials and Methods

### 2.1. Participants

A total of 160 undergraduate and graduate students from the University of Massachusetts Boston participated in the study. Our final sample consisted of 127 participants, with 37 participants classified as *low* in social anxiety (LSA: 22 females, mean age = 26.03, SD = 9.717, range = 18–54) and 90 participants classified as *high* in social anxiety (HSA; 69 cisgender females; 1 transgender individual, mean age = 23.14, SD = 6.488, range = 18–61). To be included in this final sample, participants needed to have either high or low social anxiety (see description of BFNE cut-off scores below) and not score high on a measure of depression (DASS-depression scores <17). Individuals who met qualifying scores were contacted and followed-up with a brief phone interview, used to confirm social anxiety status. See Table 1 for a summary of participant demographics. Thirty-three additional participants were excluded from this study due to an insufficient number of completed trials, with less than 30 out of 64 total trials (13); biased behavioral responses where 80% happy faces were judged happy less than 75% of the time or 80% angry faces were judged happy more than 25% of the time (9); participant error where incorrect buttons were pressed (1); technical issues (2); DASS-depression scores greater than cut off (7); or comorbid neurological conditions such as conditions which themselves alter processing of emotional information, such as autism (1). All participants were 18 years of age or older, reported normal or corrected-to-normal vision, provided written informed consent, and received a modest monetary compensation or extra credit for an approved undergraduate course. All experimental procedures and protocols were approved by the University of Massachusetts Boston Institutional Review Board (protocol # 2012148). This is an ongoing, active protocol, first approved on 21 July 2014, and most recently approved on 22 August 2025

### 2.2. Measures

The *Brief Fear of Negative Evaluation* (BFNE: [59]) is a 12-item self-report measure examining a participant’s fear of being negatively evaluated using a 1 (not at all characteristic) to 5 (extremely characteristic) Likert scale. The BFNE has been shown to have excellent internal consistency (*α* = 0.96), excellent reliability and validity [59], good test–retest reliability (*r* = 0.75), and good convergent and divergent validity [59]. We used the eight straightforward items of the BFNE (BFNE-S; [60]) to classify individuals as high in social anxiety (*HSA* group; scores >= 25) or low in social anxiety (*LSA* group; scores <= 12) based on criterion described in [61,62]. Similar cut-offs from the BFNE-S have been used in undergraduate populations [60] and clinical samples [62]. The internal consistency alpha for the BFNE-S in the current sample was 0.94.

The *Depression Anxiety Stress Scale—21-item version* (DASS; [63]) is a self-report scale with separate, reliable, and valid scales for depression, anxiety, and stress [64]. The subscales have demonstrated good internal consistency and construct validity [64]. We used the 7-item Depression subscale and excluded participants scoring above a 17 in depression (moderate depression; [63]). The internal consistency alpha for the Depression subscale in the current sample was 0.93. Given the high degree of comorbidity between social anxiety and depression [65], we aimed to isolate the effects of social anxiety by excluding individuals who scored high on the depression inventory.

The *Positive and Negative Affect Schedule—State Version* (PANAS: [66]) consists of 20 items used to assess current affective state, separated into negative affect (PANAS-NA) and positive affect (PANAS-PA) subscales, which were designed to be orthogonal [66]. For each item, participants indicate on a 5-point Likert scale how much they are experiencing each emotion (e.g., distressed, excited). We used the PANAS subscales to assess state-level affect at the beginning of the experimental protocol. The psychometric properties of the PANAS have been deemed acceptable in samples of individuals with anxiety and depression [57]. In the current sample, the internal consistency alpha for the PANAS-PA was 0.89 and 0.68 for the PANAS-NA. Please see Table 2 for a summary of participant measures.

### 2.3. Determining Perceptual Biases

#### 2.3.1. Apparatus

Visual stimuli were presented on a Nexus CRT monitor using MATLAB 2019 and the psychophysics toolbox [67,68,69]. Participants were seated 45 cm from the monitor and positioned in a chin and forehead rest to maintain a consistent viewing distance and constant visual stimuli size across participants. Responses were recorded via key press on a laptop keyboard or via a Cedrus Response Box (RB-844; Cedrus Corporation, San Pedro CA, USA). Auditory stimuli were presented via noise-cancelling headphones (3M-Peltor headset; 3M, Monroe NC, USA).

#### 2.3.2. Stimuli

We used 8 unique identities to quantify perceptual biases (4 female and 4 male; 5 White, 2 Asian, and 1 Black). We selected a subset of 100% happy, neutral, and 100% angry faces from the NimStim face database [70] with validity ratings for a given emotion of 75% or higher. Test faces were generated by morphing different proportions of an angry face and the corresponding neutral face to create an emotional continuum for each of the unique face identities (80, 40, 20, and 10% angry; neutral; and 80, 40, 20, and 10% happy). MorphMan, a software package (STOIK Imaging, Moscow, Russia), was used to morph faces by selecting points on prominent face features: eyebrow ≈ 28 mean points; eyes ≈ 30 mean points; nose ≈ 14 mean points; mouth ≈ 22 mean points; face contour ≈ 18 mean points (for sample figure showing selection points, see [71]).

We created a total of 9 test faces (4 angry morphs, 4 happy morphs, and the neutral) for each of the 8 unique face identities, for a total of 72 possible face stimuli. All faces were gray-scaled 50% and were embedded within a gray oval to eliminate non-relevant and potentially distracting stimuli such as clothing and hair. Stimuli were 595 × 595 pixels and subtended 19.8 degrees of visual angle (for sample figure, see [71]).

#### 2.3.3. Procedures

Prior to participating in the experiment, participants completed an online survey which included the BFNE and DASS assessments. For those who met the inclusion criteria for social anxiety (i.e., classified as low or high in social anxiety based on their BFNE scores), had lower levels of depression (based on DASS scores), and were interested in participating, we followed up with a phone interview to confirm their social anxiety status. The phone interview included two brief questions related to experiences with social anxiety: (1) the first question asked participants to describe how they felt in social situations, such as talking in class or going to a party where they do not know many people; and (2) the second question asked participants to describe how they felt being the focus of attention. When the respondent’s answers matched their social anxiety group, as determined from the online survey, they were invited to participate in the lab portion of the study. On the day of the experiment, prior to starting the task, participants were asked to complete the PANAS subscales, which assessed their current state affect. Finally, to ensure that participants were familiarized with the experimental procedure, participants completed a minimum of two practice trials. Practice trials consisted of an auditory alerting cue, followed by a blank oval (1 s) and then a question mark (1.5 s), during which time, participants had to press a button to practice the timing of when to indicate their judgment.

**Figure 1:**
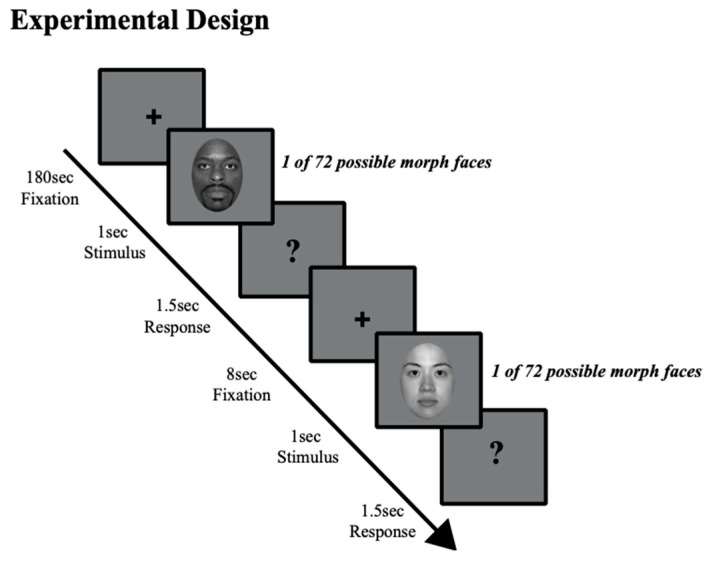
Experimental design. Participants judged a series of randomly selected faces as either happy or angry. Possible face stimuli included 8 unique face identities—4 male and 4 female faces—with each identity morphed along an emotional continuum from angry to neutral to happy (72 possible face stimuli). A fixation cross appeared at screen center (180 s), which participants were asked to fixate. Then, a brief auditory cue (500 Hz) alerted participants to an upcoming face morph (1 s), followed by a question mark (1.5 s). While the question mark was displayed, participants judged if the previously displayed face morph was happy or angry. After the question mark, a fixation cross appeared at screen center (8 s), and the sequence described above was repeated for the next trial. A total of 64 possible trials were presented.

Participants were instructed to fixate their gaze on the center of a blank gray screen for the duration of the experiment, but eye position was not monitored. A trial started with a fixation cross at screen center (180 s), followed by an auditory cue (500 Hz). The auditory cue alerted participants that the face stimulus would soon be presented and would need to be judged. Then, one face morph, out of 72 possible morphs, was presented at random (1 s), followed by a question mark (1.5 s), during which time participants had to judge the emotion of the face that had just been presented. Participants pressed one key if they judged the face to be happy and another key if they judged it to be angry. At the end of each trial a blank screen with a fixation cross was presented (8 s). A total of 64 trials were presented: 8 trials for each happy and each angry face morph (40%, 20%, 10%), 8 neutral, and 4 trials for each happy and angry face morph (80%). Trials in which participants responded before or after the 1.5 s response period when the question mark was presented were excluded. Details of the experimental design are shown in Figure 1. These methods are adapted from those in a related study in a non-clinical cohort [50]. Of note, the experimenter conducting the study was masked as to the social anxiety status of the participant.

### 2.4. Data Analysis

#### 2.4.1. Quantifying Biases in the Perception of Emotional Faces

All single-subject data were fit using MATLAB 2019 (The MathWorks, Inc., Natick, MA, USA). We used *psignifit* (https://psignifit.sourceforge.net/, accessed on 27 October 2025), which implements the maximum-likelihood method described by Wichmann and Hill [72], to fit the data for each participant to determine their PSE, a standard measure in visual psychophysics. We plotted the emotional morph continuum along the *x*-axis and the percentage of trials the participant judged a given face morph as happy on the *y*-axis and fit the data to a cumulative normal function. The PSE quantifies the percent of emotion in a face required to perceive it as equally likely to be judged as happy or angry or at chance. We plotted happy emotions as having positive values (to the right of 0 on the *x*-axis) and angry emotions as having negative values (to the left of 0 on the *x*-axis). Thus, a positive PSE indicates more happiness is required to perceive a face as equally happy or angry, indicating a negative perceptual bias. Conversely, a negative PSE indicates more anger is required to see a face as equally happy or angry, indicating a positive perceptual bias.

**Figure 2:**
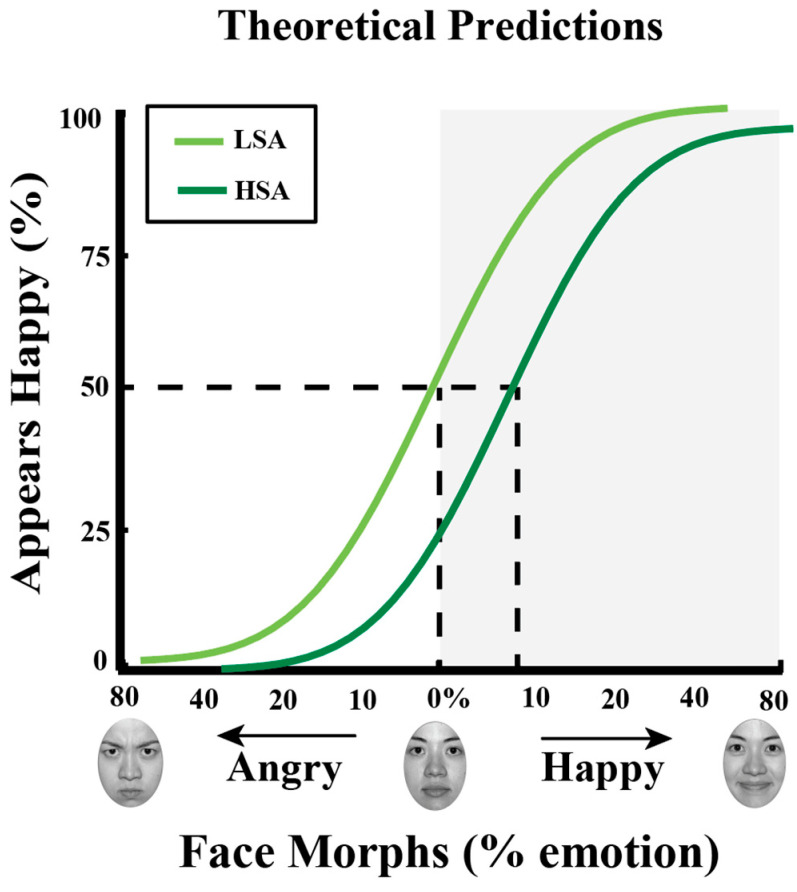
Theoretical predictions for unique neutral (PSE). The *x*-axis represents the morph face continuum from angry to happy, with 0 representing the standard neutral as defined by the NimStim dataset. The *y*-axis represents the percentage of happy responses. Data points were fit using a cumulative normal function. The dark green line shows the fit to face morphs for individuals high in social anxiety (HSA) and the light green line for individuals low in social anxiety (LSA). We quantified each participant’s PSE by determining the face morph supporting 50% happy judgments, i.e., the morph equally likely judged happy as angry. A more positive PSE indicates more happiness is needed to judge a face as equally happy or angry; thus, the face is judged angrier, reflecting a negative bias. We predicted individuals low in social anxiety would show a weaker negative bias (a less positive PSE even negative PSE) while individuals high in social anxiety would show a stronger negative bias (a more positive PSE). In this hypothetical example, the PSE is 0% angry and 8% happy for individuals low versus high in social anxiety, respectively. Such a hypothetical example illustrates that faces are biased to be perceived more negatively in high relative to low social anxiety.

To illustrate the transformation of continuous, parametric data into a single value, the PSE, we show fits for hypothetical data and the resulting PSE (see Figure 2). In this hypothetical example, the PSE is at 8% happy for the individual high in social anxiety, indicating that a face needs to contain more happiness to be judged equally happy or angry, i.e., a negative bias.

For each participant, we also estimated the slope of the psychometric function at the PSE. A steeper slope would indicate a more categorical representation of happy and angry, whereas a shallower slope would indicate a more gradual change in judgments of happy and angry faces. The slope value provides another measure by which to assess perceptual bias in terms of the rate of change in the categorization of faces across an emotional continuum. Thus, even if the PSE measure does not differ by anxiety group, the slope measure may still differ by anxiety group, with a steeper slope suggesting less ambiguity and clearer categorical boundaries in rating faces as happy or angry.

#### 2.4.2. Statistical Analyses

Our primary dependent variables were PSE and slope at the PSE, and our primary independent variable was social anxiety status (high versus low). We also considered negative affect as a moderator. Our dependent measures of PSE and slope were normally distributed, with skew between +1 and −1 and kurtosis between +2 and −2 [73]. Despite our unequal sample sizes between groups low and high in social anxiety, Levene’s test of equality of variances found no significant difference in variance across groups for our main measure of PSE. Given that our measure of NA was slightly skewed, we applied a square root transformation, bringing the measure within acceptable limits, and used transformed data for subsequent analyses.

We ran independent samples t-tests to determine if our main dependent measures of PSE and slope were significantly different between our high and low social anxiety groups. Furthermore, two separate hierarchical linear regression models with robust standard errors were used to estimate the influence of social anxiety status on PSE and slope. To test the hypothesis that high social anxiety would predict greater PSE, particularly for those with greater levels of state affect, a moderation analysis was conducted using Hayes’ PROCESS macro where social anxiety group and state affect, as well as their interaction, were entered into the model to predict PSE [74].

All analyses were two-tailed, and statistical significance was defined as *p* < 0.05. Effect sizes for *t*-tests were determined using Cohen’s d. Analyses were conducted using IBM SPSS Statistics (Version 26). Data fitting to determine PSE and slope and to create figures was carried out using MATLAB 2019 (The MathWorks, Inc., Natick, MA, USA).

## 3. Results

### 3.1. Perceptual Bias, as Assessed by the PSE

Our final sample of 127 participants completed a total of 7,495 trials. Trials were excluded if participants did not judge the emotion of the face during the 1.5 s response interval. Of 64 total trials, LSA participants completed an average of 57.76 trials (SD = 7.25) and HSA participants an average of 59.76 trials (SD = 6.42). There were no significant differences in the number of trials completed by HSA and LSA individuals (independent samples *t*-test, two-sided; t (125) = −1.319, *p* = 0.19, d = 0.258). As expected, we found significantly higher scores of depression in HSA individuals (mean DASS = 8.63) compared to LSA individuals (mean DASS = 5.51; independent samples t-test, two-sided; t (124) = −3.377, *p* < 0.001, d = 0.661). Furthermore, negative mood (NA) was significantly higher in HSA individuals (mean square root NA = 3.69) compared to LSA individuals (mean square root NA = 3.41; independent samples t-test, two-sided; t (102.726) = −4.280, *p* < 0.001, d = 0.398; equal variance not assumed). Of note, while there was a significant difference in negative affect (NA) between those high and low in social anxiety, the effect size was small, and both the LSA and HSA groups showed variability in NA measures, with 18.9% of LSA participants and 14.1% of HSA participants exhibiting negative affect levels 1SD above the mean. This small effect size also indicates the multicollinearity between these variables is not a problem.

**Figure 3:**
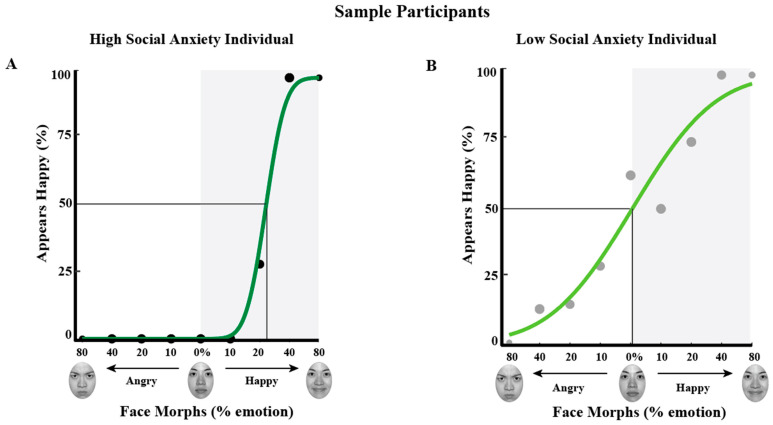
Biases in PSE in sample individuals low vs. high in social anxiety. Data are plotted and fit with a cumulative normal to estimate PSE for a sample participant high in social anxiety ((**A**): dark green) and low in social anxiety ((**B**): light green). The *x*-axis depicts the face morph continuum, which ranges from 80% angry to 80% happy for a sample probe face. The gray shaded area highlights happy face morphs, with 0 indicating the standard neutral face, as defined by the NimStim dataset. The *y*-axis depicts percent happy judgments. Chance performance is at 50%, with values less than 50% indicating an angry judgment. The black curve is a fit to the data, with each data point showing average percent happy judgments for a given face morph. For the participant with high social anxiety (**A**), the PSE was 21.95% happy. For this participant, to see a face as equally happy or angry required ~22% more happiness in the face, representing a negative bias. For the participant with low social anxiety (**B**), the PSE was 0.56% happy. For this participant, there was no overall bias to see faces as angry; faces required not even 1% more happiness to be perceived as equally happy or angry. Furthermore, for these two sample participants, the slope of the fit at the PSE is steeper in the HSA (slope = 0.76) compared to the LSA individual (slope = 0.19), indicating a more categorical judgment of happy and angry faces in the HSA individual.

To illustrate data from individual participants and provide an example of what might be experienced in each group and how that is depicted in our measure, PSE measures are shown for a single LSA and a single HSA participant (Figure 3A and Figure 3B, respectively). For the LSA individual (Figure 3B), faces judged 50% happy contained 0.56% happiness (PSE = 0.5615). For the HSA individual (Figure 3A), faces judged 50% happy contained 21.95% happiness (PSE = 21.95). This indicates a negative bias in the HSA individual: the face judged equally likely happy or angry required more than 20% more happiness in the participant high in social anxiety. Furthermore, for these two sample participants, the slope of the fit at the PSE is much steeper in the HSA (slope = 0.76) compared to the LSA individual (slope = 0.19), indicating a more categorical judgment of happy and angry faces in the HSA individual.

### 3.2. The Influence of Social Anxiety and Negative Affect on PSE

**Figure 4:**
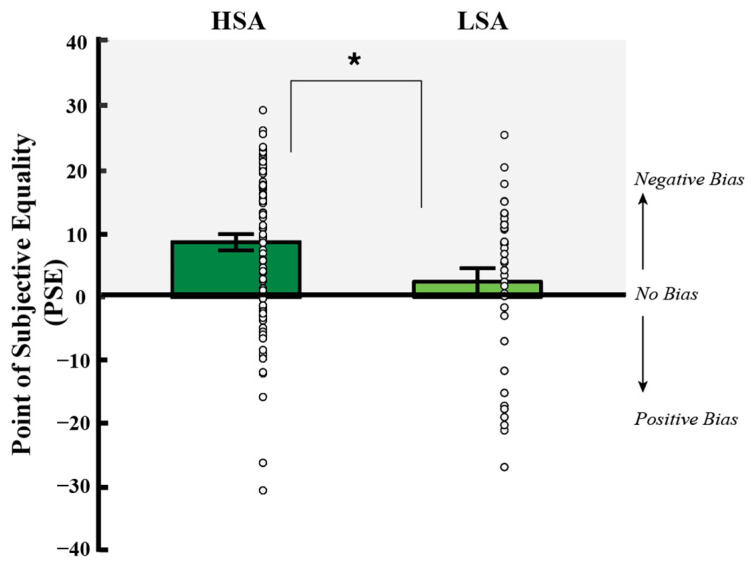
Biases in PSE at baseline as a function of social anxiety (mean +/− SEM across participants). Perceptual biases, as assessed by unique neutral, or PSE, the percent morph judged emotionally neutral, are displayed by social anxiety group: high social anxiety (HSA) and low social anxiety (LSA). The *y*-axis depicts mean PSE (+/−SEM across participants, with individual data shown as circles). A value of 0 represents no perceptual bias (PSE equals standard neutral), and a positive value indicates a negative perceptual bias, since more happiness is needed to see a face as equally happy or angry. Data are shown for individuals high (left; dark green) and low (right; light green) in social anxiety. Mean PSE was 8.69 (SD = 12.17) in HSA and 3.04 (SD = 11.88) in LSA. HSA individuals had a significantly more positive PSE compared to LSA individuals. * indicates *p* < 0.05.

Perceptual biases, quantified by PSE, are shown as a function of social anxiety status in Figure 4 (mean PSE in HSA = 8.69 (SD = 12.17); mean PSE in LSA = 3.04 (SD = 11.88)). We used a hierarchical regression model to assess if social anxiety and negative affect predict our main dependent measure of PSE. In Step 1, social anxiety status and negative affect (NA) were entered as predictors (Model 1). In Step 2, the interaction term (Social Anxiety Status x NA) was entered as an additional predictor (Model 2). Results are shown in Table 2.

Model 2 provided a better fit: R squared was larger for Model 2 versus for Model 1, 0.10 versus 0.06, respectively, with a significant ΔR^2^, as elaborated on below. Model 2 showed that when both variables are in the model, social anxiety was a significant unique predictor of PSE (β = 0.29, *p* = 0.01), while negative affect did not uniquely predict PSE (β = −0.35, *p* = 0.15). A post hoc t-test revealed a more positive PSE in HSA compared to LSA individuals (t (125) = −2.394, *p* = 0.018, d = 0.468) of moderate effect size. Thus, for a face to appear equally happy or angry, the PSE had to contain more happiness, a negative perceptual bias, in individuals high compared to those low in social anxiety.

### 3.3. Interaction Between Social Anxiety Status and Negative Affect on PSE

**Figure 5:**
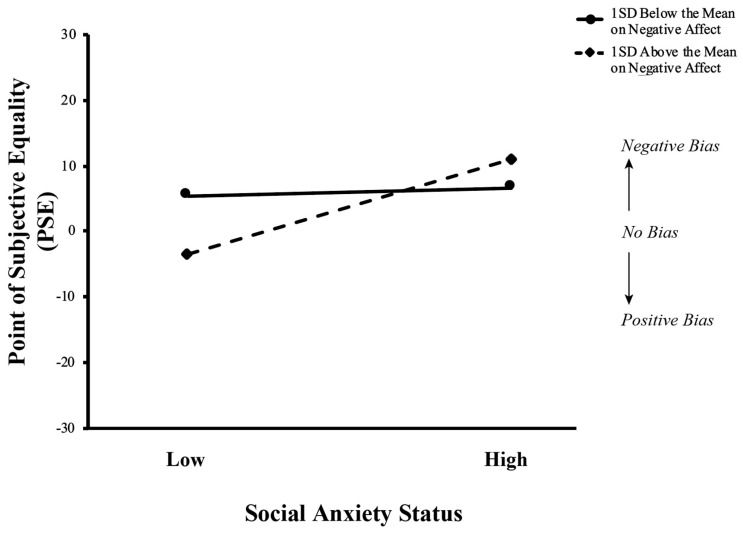
The interaction between social anxiety status and negative affect on perceptual bias. Negative affect significantly moderated the relationship between social anxiety status and point of subjective evaluation (PSE). PSE is plotted at different levels of the moderator negative affect (−1 and +1 standard deviations from the mean) for low social anxiety and high social anxiety groups.

The first moderation model examined the interaction of social anxiety status and negative state affect (social anxiety status x NA) in predicting PSE, with results summarized in Table 3. The interaction between social anxiety status and negative state affect accounted for a significant proportion of the variance in PSE (ΔR^2^ = 0.03, F (1, 118) = 4.21, *β* = 15.67, t (118) = 2.05, *p* < 0.05), such that NA significantly moderated the association between social anxiety status and PSE. The interaction term was a significant predictor of PSE (β = 0.47, *p* = 0.04). This interaction is represented in Figure 5. In examining the simple slopes, there was no significant change between those low and high in social anxiety for individuals at one standard deviation below the mean for negative affect (t = 0.36, *p* = 0.72); however, for those at one standard deviation above the mean on negative affect, the simple slope was significant (t = 2.78, *p* = 0.01), such that those low in social anxiety had significantly lower PSEs than those high in social anxiety.

### 3.4. The Influence of Social Anxiety and Negative Affect on Slope

**Figure 6:**
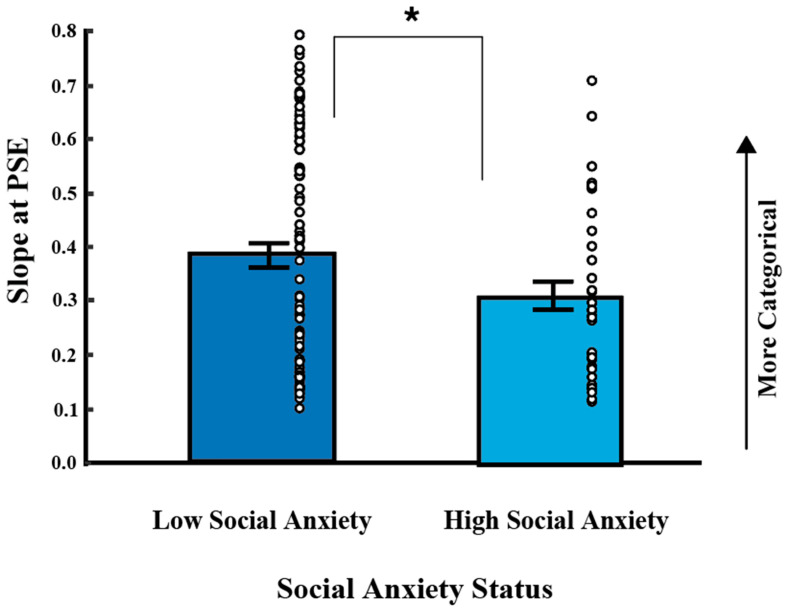
Slope as a function of social anxiety (mean +/− SEM across participants). Slope is displayed as a function of social anxiety status: high social anxiety (HSA) and low social anxiety (LSA). The *y*-axis depicts mean slope (+/−SEM across participants, with individual data shown as circles). Increasing levels of slope indicate more categorical perception of the emotional faces. Data are shown for individuals high (left; dark blue) and low (right; light blue) in social anxiety. Mean slope was 0.384 (SD = 0.213)) in HSA and 0.290 (SD = 0.154)) in LSA individuals. Slopes were significantly greater in HSA compared to LSA individuals. * indicates *p* < 0.05.

Figure 6 shows slope measures in HSA (mean = 0.384 (SD = 0.213)) and LSA (mean = 0.290 (SD = 0.154)) individuals. We used a hierarchical regression model to assess how social anxiety and negative affect predict our main dependent measure of slope. In Step 1, social anxiety status and negative affect (NA) were entered as predictors. In Step 2, the interaction term (social anxiety status x NA) was entered as an additional predictor. Results are shown in Table 4. In the full model, social anxiety was a significant unique predictor of slope (β = 0.22, *p* = 0.02), while negative affect did not uniquely predict slope (β = −0.24, *p* = 0.13). No statistically significant interaction was observed between social anxiety status and negative affect for slope (ΔR^2^ = 0.07, F (3, 118) = 2. 82, b = 0.06, t (118) = 1.44, *p* = 0.15), as summarized in Table 4. Thus, negative affect does not moderate the relationship between social anxiety and slope. A post hoc t-test reveals a more positive slope in HSA compared to LSA individuals (t (125) = −2.44, *p* = 0.016, d = 0.477) of moderate effect size. This suggests that participants high in social anxiety have a more categorical perception of angry versus happy faces.

## 4. Discussion

We examined how *perceptual* biases for emotional stimuli were influenced by social anxiety and current affective state. To this end, we quantified the amount of emotion required in faces for them to be judged equally often as happy or angry by calculating each participant’s unique point of subjective equality, PSE. We expected perceptual bias to be more negative for individuals high in social anxiety, as reflected by a more positive PSE, and for negative perceptual bias to be more pronounced with more negative state affect.

We found a significant main effect of social anxiety on perceptual biases, such that individuals with high social anxiety demonstrated greater negative perceptual bias. This effect of social anxiety on perceptual bias was qualified by a significant interaction with state affect. In accordance with our hypothesis, stronger negative state affect (larger NA) was associated with stronger negative biases (more positive PSE), a *mood-congruent effect*, particularly, in individuals high in social anxiety. Thus, despite not inducing state affect in our experiment, the current state affect someone brings with them to the experiment is important to consider. Interestingly, this pattern was not observed in individuals low in social anxiety. *This suggests an interesting compensatory effect in LSA participants, where internalized negative state affect experienced at a given moment in time may not translate into a negative perceptual bias—such a mechanism may help maintain lower levels of trait anxiety.* Our results highlight the possibility that differences in perceptual bias across social anxiety groups may be modified by underlying differences in negative state affect across individuals and across groups high versus low in social anxiety; such effects could contribute to disparate results across studies.

### 4.1. Biases in Negative Affect and Biases in Perception

A growing body of work highlights the importance of considering not only trait affect but also state affect, including studies inducing mood or quantifying non-induced mood. Studies find that inducing mood can bias emotional perception. For example, faces are judged more negatively following induction of a depressive mood and more positively following induction of a happy mood, a *mood-congruent effect* [75]. Furthermore, in a dynamically changing emotional faces paradigm, the emotion perceived in a face persisted longer for congruent versus incongruent moods. In other words, happy mood induction resulted in a face being perceived as happy for longer when it was dynamically morphing from happy to neutral [76]. These results suggest that induced mood can enhance sensitivity for perceiving congruent facial emotions. Interestingly, inducing a negative mood can bias the deployment of exogenous attention to positive faces [77,78], yielding compensatory mechanisms, which might potentially mitigate the consequences of the negative mood.

State affect (one’s current, non-experimentally induced mood) has also been found to bias perception in non-clinical cohorts [50,51]. The current study examines the influence of state affect in biasing perception as a function of social anxiety status specifically. We find differential effects of negative state affect. Only HSA individuals showed *mood-congruent effects*: a more negative mood correlated with more negative perceptual bias, a positive feedback mechanism which may work to perpetuate the negative mood and negative perceptual bias.

Our results lend insight into potential differences in emotional regulation and cognitive strategies in high versus low social anxiety. Our findings of mood-congruent effects in HSA but not LSA individuals may reveal core differences in how social anxiety interacts with mood to either protect against socially anxious symptoms or promote them. Future research will need to delineate differences in how attention and perception are altered by state affect in individuals along the social anxiety spectrum. It remains to be seen whether the self-perpetuating mechanism observed in high social anxiety bears any resemblance to mechanisms resulting from mood changes which are experimentally induced.

### 4.2. Considerations of State Affect Versus Trait Anxiety

We found an interesting interaction between state affect and trait social anxiety on perceptual biases: a positive correlation between state affect and perceptual bias, but only in individuals high, but not low, in social anxiety. This extends the work of Quigley and colleagues [38], who found that state affect increased attentional bias regardless of trait anxiety, highlighting the importance of examining both state and trait affect and their interaction. Our finding is similar to previous work showing that state anxiety status modulates the effects of trait anxiety on cognitive processing [46,47]. In this way, it is both one’s disposition towards anxiety, as well as one’s current mood, that influences perceptual biases.

### 4.3. Limitations

As with all research, this study has several limitations. The current study design cannot speak to the role of overt orienting of attention in biasing perceptual processing. While we asked participants to maintain central fixation, eye position was not monitored. Thus, differences in the scanning of different features of a face as a function of social anxiety and contributions of such differences to perceptual biases are unclear.

Of note, our results are from a student sample categorized along the continuum of social anxiety via the BFNE. BFNE cutoff scores are suggestive of clinically significant social anxiety, e.g., [61], but it remains to be seen how these effects apply more generally to clinical cohorts. One possible consequence of using a non-clinical population is that differences in PANAS may underestimate the strength of correlations between perceptual biases and state affect. Furthermore, we minimized the influence of depression in our study by only including participants with a DASS score of less than or equal to 17. Yet, depression and social anxiety are often comorbid, and our observed effects may have been stronger if the covariate of depression was more influential. While we controlled for the influence of depression, we did not control for stress. It is possible that the trait level of a more general stress and anxiety response is influencing the results of this study, and this should be considered in future studies. Future studies will need to replicate and extend these findings to consider mechanisms that may enhance negative perceptual bias in HSA individuals with stronger negative affect while weakening negative perceptual bias in LSA individuals with stronger negative affect.

There is also a need to examine a broader range of stimuli conveying negative affect, especially for non-threatening emotions such as sadness. Of note, in previous work, we found interesting differences in the strength of adaptation effects in individuals high versus low in social anxiety but only for threatening negative emotions, i.e., angry, and not for non-threatening negative emotions, i.e., sadness [58]. Furthermore, future studies should examine the impact of inducing negative affect in social anxiety (e.g., anticipation of a speech) on the studied constructs. The effects we observed may have been more robust if we had experimentally induced negative state affect, such as by requiring participants to give a public speech, which might have led to larger NA scores, as this is a stressful situation, or if we had a broader range of negative affect, if we had not restricted DASS to values less than or equal to 17. It would also be important to consider the specificity of these findings to social anxiety by considering other clinical presentations that show attention/perception biases (e.g., dysphoric or depressed individuals), as well as considering the influence of other covariates, such as age, race, and gender, in a larger study with enough power to assess such influences. We also did not consider the influence of other covariates such as measures of income, education, or socioeconomic status. Finally, our study considered the effects of negative affect as a between-subject measure. Negative affect should also be considered using within-person comparisons, i.e., comparing perceptual biases when participants feel more negative affect to when they feel less negative affect. In general, we have not found exposure to the emotional faces used in our study to have a strong influence on state affect (unpublished data), as participants are seated in a comfortable room viewing stimuli on a screen compared to situations such as anticipation of a speech in public.

### 4.4. Theoretical Implications

The current study contributes to models of social anxiety by indicating that perceptual biases of emotions are not just a function of trait vulnerability but also interact with transient affective states such as negative affect. Prominent cognitive models of social anxiety highlight the role that negative biases play in maintaining symptoms of social anxiety [9]. However, our results suggest that such biases may be conditional, with negative affect amplifying negative perception of emotional faces only in individuals with high social anxiety but not in those with low social anxiety. The absence of such effects in low socially anxious individuals may reflect compensatory mechanisms that buffer against mood-congruent cognitive biases. Our findings suggest that perceptual biases in social anxiety are dynamic rather than fixed and that they emerge from the interplay between trait anxiety and momentary affective states.

### 4.5. Practical Implications

From an applied perspective, the results of our study suggest that therapeutic interventions for social anxiety may benefit from targeting not only enduring, persistent, cognitive biases but also momentary mood states. For instance, some therapeutic approaches teach individuals to recognize, understand, and regulate negative affect [79] (for a review, see [80]), which can help mitigate the amplification of perceptual biases during stressful social interactions or situations. Thus, taking into account the interactive role of current state affect could potentially strengthen the assessment and further management of social anxiety symptoms. Relying on trait measures alone may overlook the importance of fluctuations in perceptual biases due to current mood or state affect. For instance, evidence from ecological momentary assessment studies suggests that individuals with increased levels of social anxiety experience significant psychological benefits, including increased happiness, reduced avoidance motivation, and a sense of belonging, following positive events in the past hour [54]. Together with our findings, this evidence highlights the importance of therapeutic approaches that simultaneously decrease negative perceptual biases and cultivate positive experiences.

## 5. Conclusions

In summary, the present study provides evidence that biases in perceiving emotional faces are shaped by an interaction between social anxiety and current affective state, specifically negative affect. While individuals with high levels of social anxiety exhibited mood-congruent perceptual biases such that higher negative affect was linked to more negative perceptual biases, those with low levels of social anxiety exhibited the opposite effect, suggesting a protective mechanism against negative state affect spillover into perception of emotional information. Our findings extend the existing models of emotion perception, underscoring the need to integrate trait and state anxiety in both theory and practice, which ultimately points to new directions for intervention in social anxiety.

## Figures and Tables

**Table 1:** Participant demographics (*n* = 127).

	Low Social Anxiety (*n* = 37)	High Social Anxiety (*n* = 90)
Demographic characteristics		
Age, mean (SD; range)	26.03 (9.72; 18–54)	23.14 (6.29; 18–61)
Gender, n (%)	20 (54.05%) female 17 (45.95%) male	70 (77.78%) female 19 (21.11%) male 1 (1.11%) transgender
Race/ethnicity n (%)		
White	19 (51.35%)	47 (52.22%)
Latino/Hispanic	6 (16.22%)	5 (5.56%)
Asian American	1 (2.70%)	18 (20.00%)
Black/African American	8 (21.62%)	11 (12.22%)
Multiracial	0 (0.00%)	3 (3.33%)
Unspecified	3 (8.11%)	6 (6.67%)

**Table 2:** Participant trait and state affect measures (*n* = 127).

	Low Social Anxiety (*n* = 37)	High Social Anxiety (*n* = 90)
**Clinical characteristics** **Mean (SD; range)**		
BFNE	10.14 (1.55; 8–12)	32.07 (4.39; 25–40)
DASS-Depression	5.41 (4.65; 0–14)	8.63 (4.97; 0–17)
PANAS-Positive Affect (PA)	32.73 (8.15; 19–50)	27.28 (8.19; 11–46)
PANAS-Negative Affect (NA)	11.68 (2.02; 10–18)	13.81 (3.38; 10–26)

Notes: SD = standard deviation; BFNE = Brief Fear of Negative Evaluation; DASS= Depression Anxiety Stress Scales—21-item version; PANAS= Positive and Negative Affect Schedule. Note: positive and negative affect values are missing from 5 participants.

**Table 3:** Summary of hierarchical regression analysis of the interaction of social anxiety status and negative affect on point of subjective equality.

Variable	*β*	*t*	*R* ^2^	Δ*R*^2^	*p*
Model 1			0.06	0.06	0.021
Social anxiety status	0.20	2.11			0.037
Negative affect (NA)	0.10	1.12			0.267
Model 2			0.10	0.03	0.042
Social anxiety status	0.29	2.82			0.006
Negative affect (NA)	−0.35	−1.45			0.149
Social anxiety status x NA	0.47	2.05			0.042

Note. NA = negative affect.

**Table 4:** Summary of hierarchical regression analysis of the interaction of social anxiety status and negative affect on slope.

Variable	*β*	*t*	*R* ^2^	Δ*R*^2^	*p*
Model 1			0.05	0.05	0.045
Social anxiety status	0.22	2.44			0.016
Negative affect (NA)	−0.05	−0.57			0.569
Model 2			0.07	0.02	0.154
Social anxiety status	0.22	2.43			0.017
Negative affect (NA)	−0.24	−1.51			0.134
Social anxiety status × NA	0.23	1.44			0.154

Note. NA = negative affect.

## Data Availability

Data and analysis code are available on Open Science Framework via this link: https://osf.io/bjypt/. The study design and analysis were not preregistered. Here, we used all baseline data that was collected as part of a bigger study on adaptation to emotional faces. Data from a subset of these adaptation studies have been reported in related work (Morina, Harris, Hayes-Skelton, & Ciaramitaro, 2024) [58]. The adaptation data only included a subset, as not all participants who completed the baseline data block completed the adaptation data block. Sample size was determined for these adaptation studies from which this baseline data was collected.

## Abbreviations

The following abbreviations are used in this manuscript:

PSE	point of subjective equality
LSA	low in social anxiety
HSA	high in social anxiety
BFNE	Brief Fear of Negative Evaluation
DASS	Depression Anxiety Stress Scales
PANAS	Positive and Negative Affect Schedule
PA	positive affect
NA	negative affect
